# Fast gene disruption in *Trichoderma reesei* using in vitro assembled Cas9/gRNA complex

**DOI:** 10.1186/s12896-018-0498-y

**Published:** 2019-01-09

**Authors:** Zhenzhen Hao, Xiaoyun Su

**Affiliations:** Key Laboratory for Feed Biotechnology of the Ministry of Agriculture, Feed Research Institute, Chinese Academy of Agricultural Sciences, No. 12 South Zhongguancun Street, Beijing, 100081 China

**Keywords:** CRISPR/Cas9, *Trichoderma reesei*, Gene disruption, Ribonucleoprotein

## Abstract

**Background:**

CRISPR/Cas9 has wide application potentials in a variety of biological species including *Trichoderma reesei*, a filamentous fungus workhorse for cellulase production. However, expression of Cas9 heterologously in the host cell could be time-consuming and sometimes even troublesome.

**Results:**

We tested two gene disruption methods in *T. reesei* using CRISPR/Cas9 in this study. The intracellularly expressed Cas9 led to unexpected off-target gene disruption in *T. reesei* QM9414, favoring inserting 9- or 12-bp at 70- and 100-bp downstream of the targeted *ura5*. An alternative method was, therefore, established by assembling Cas9 and gRNA in vitro, followed by transformation of the ribonucleoprotein complex with a plasmid containing the *pyr4* marker gene into *T. reesei* TU-6. When the gRNA targeting *cbh1* was used, eight among the twenty seven transformants were found to lose the ability to express CBH1, indicative of successful *cbh1* disruption through genome editing. Large DNA fragments including the co-transformed plasmid, chromosomal genes, or a mixture of these nucleotides, were inserted in the disrupted *cbh1* locus.

**Conclusions:**

Direct transformation of Cas9/gRNA complex into the cell is a fast means to disrupt a gene in *T. reesei* and may find wide applications in strain improvement and functional genomics study.

**Electronic supplementary material:**

The online version of this article (10.1186/s12896-018-0498-y) contains supplementary material, which is available to authorized users.

## Background

*Trichoderma reesei* is a well-known filamentous fungus workhorse for production of cellulase and other industrially important proteins [1]. Gene disruption is a critical technique for functional genomics study of *T. reesei*. From a practical perspective, deleting key regulatory genes that repress cellulase expression has proved to be very useful in strain improvement [2]. In addition, removal of major cellulase and protease genes is an essential step towards creating a platform strain for heterologous protein production [3]. Therefore, a method is needed to efficiently disrupt the genes of interest in *T. reesei*.

In *T. reesei*, gene disruption is routinely achieved by gene replacement through homology-based DNA recombination (HDR) [4]. However, one should note that the efficiency of HDR in *T. reesei* is notoriously low. Knocking out *ku70* or *mus53* involved in non-homologous end-joining (NHEJ) can improve the HDR rate [5], but time is needed to obtain a recipient strain with abated NHEJ function. The CRISPR/Cas9 system (with CRISPR standing for *c*lustered *r*egularly *i*nterspaced *s*hort *p*alindromic *r*epeats) is the bacterial type-II adaptive immune system [6], which can serve as an alternative means for gene disruption in *T. reesei*. In this system, the gRNA recognizes specific target DNA sequence, forms DNA/RNA duplex at the locus of recognition, and guides the endonuclease Cas9 to cleave the DNA. The CRISPR/Cas9 technology has been widely used for genome editing in mammalian cells, plants, and microbes. Genome disruption using CRISPR/Cas9 has been reported for a variety of filamentous fungi [7], which commonly requires expression of *cas9* in vivo. In contrast, transformation of the in vitro assembled Cas9 and gRNA complex provides a fast means of genome disruption [8], which however has not been reported in *T. reesei*.

In the present study, it was first found that the intracellularly expressed Cas9 led to unexpected off-target gene disruption in *T. reesei* QM9414. An alternative means, i.e. direct transformation of the in vitro assembled Cas9/gRNA, was evaluated for its efficacy in gene disruption using the cellobiohydrolase I (*cbh1*) gene as a model. The sequences of the disrupted gene were analyzed, identifying insertion of large chromosomal DNAs or plasmid fragments.

## Results

### Intracellularly expressed Cas9 led to unexpected off-target genome editing

The *cas9* gene has been expressed constitutively or induced successfully in *T. reesei*, in both cases which can be used in successful genome editing in QM6a and RUT-C30 [7]. The codon-optimized *cas9* gene [7] was synthesized, ligated downstream of the strong, constitutive *pdc1* promoter, and transformed into *T. reesei* QM9414. Nine positive transformants were obtained, among which strain C5 had the highest *cas9* transcript level (determined by RT-qPCR, data not shown) and was therefore selected for subsequent gene disruption.

**Fig. 1:**
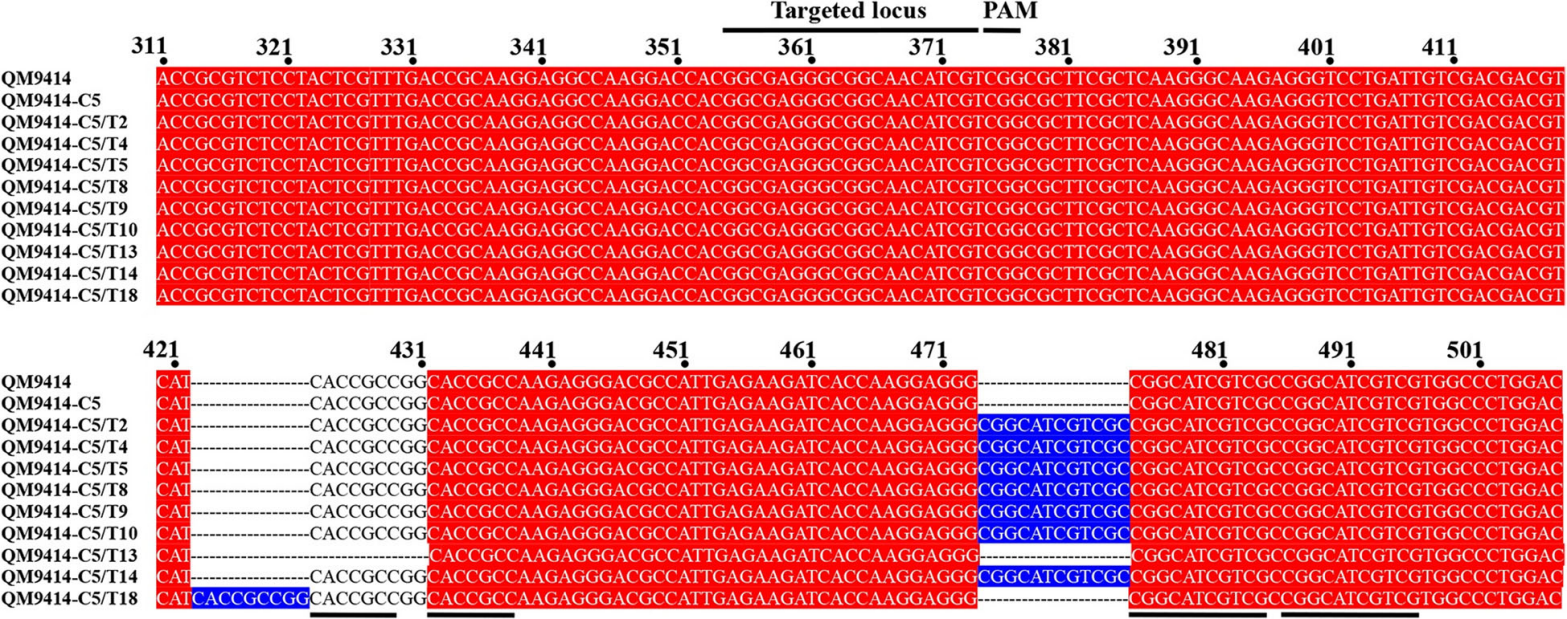
Intracellularly expressed Cas9 led to unexpected insertion and deletion in *ura5.* The inserted oligonucleotides were labeled in blue and the direct repeats were underlined

The *ura5* gene has been used as the target when the CRISPR/Cas9 technology was established in *T. reesei* [7]. The in vitro transcribed gRNA targeting the same *ura5* locus was transformed into C5. Nineteen 5-fluoroorotic acid (5-FOA) resistant transformants were obtained. While no mutation was found in the expected editing locus for all these transformants, seven had an unexpected insertion of 12-bp at the 100-bp downstream of the expected editing locus (Fig. 1, Additional file 1: Figure S1). One (QM9414-C5/T18) had an insertion of 9-bp, while another (QM9414-C5/T13) had a deletion of this 9-bp fragment, at the 70-bp downstream of the expected editing locus. Interestingly, the inserted short DNA fragments were a direct repeat of the nucleotides downstream of the insertion site. Moreover, two direct repeats (7- and 11-bp, respectively) were found in both sites (Fig. 1), suggestive of biased off-target editing.

### Designing gRNA for *cbh1* gene disruption

**Fig. 2:**
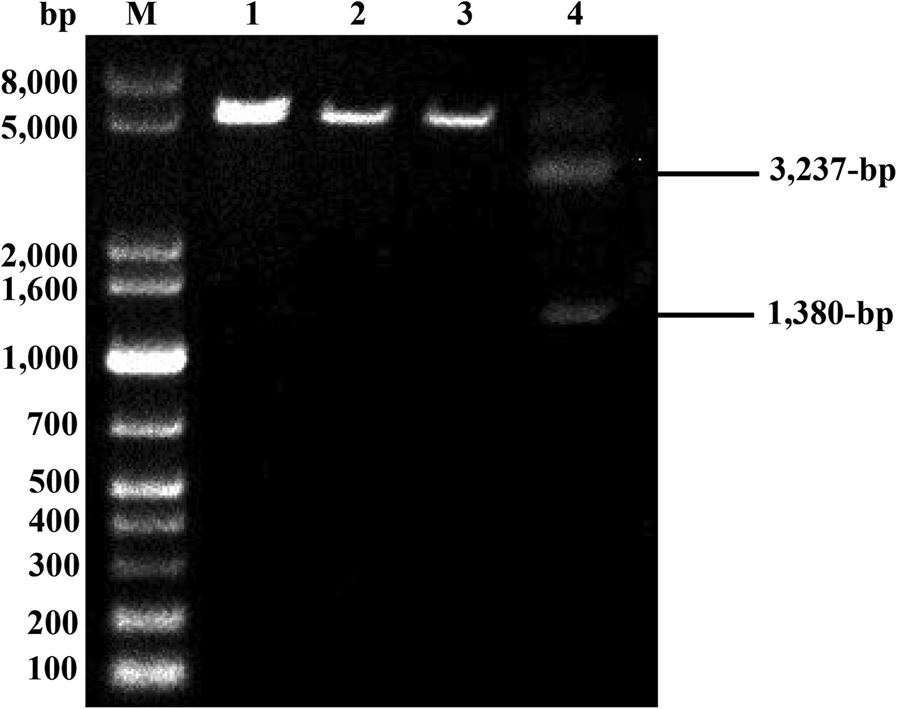
In vitro digestion of *cbh1* by Cas9 and gRNAs. M: DNA molecular mass marker; lane 1: *Bcu*I-linearized plasmid; lane 2–4: *Bcu*I-linearized plasmid incubated with Cas9 and gRNA1 (lane 2), gRNA2 (lane 3), and gRNA3 (lane 4)

Direct transformation of Cas9/gRNA complex may be an alternative for genome editing. Indeed, this technique has already been successfully used for gene disruption in various filamentous fungi including *Mucor circinelloides* [9], *Aspergillus fumigatus* [10], and *Fusarium oxysporum* [11]. For those filamentous fungi that are difficult to manipulate, the transformantion of Cas9/gRNA complex provides a means to speed up genome editing. With this advantage, nevertheless, there was no report of using such a technique in *T. reesei*. CBH1 is the major cellulase secreted by *T. reesei*, accounting for above 50–60% in the fermentation broth [12]. Disruption of *cbh1* will result in loss of the major band on SDS-PAGE gel, facilitating identification of the strains with successful genome editing. Therefore, for ease of verification, *cbh1* was selected as the target gene. Three different gRNAs on *cbh1* were designed and synthesized by in vitro transcription. Cas9 and a certain gRNA was incubated with the *Bcu*I-linearized pT3cbh1 plasmid (4717-bp) containing the *cbh1* gene. gRNA-3 was the best one to guide Cas9-mediated digestion of pT3cbh1 into two DNA fragments with expected sizes (Fig. 2).

### Disruption of *cbh1* involved insertion of large DNA fragments

**Fig. 3:**
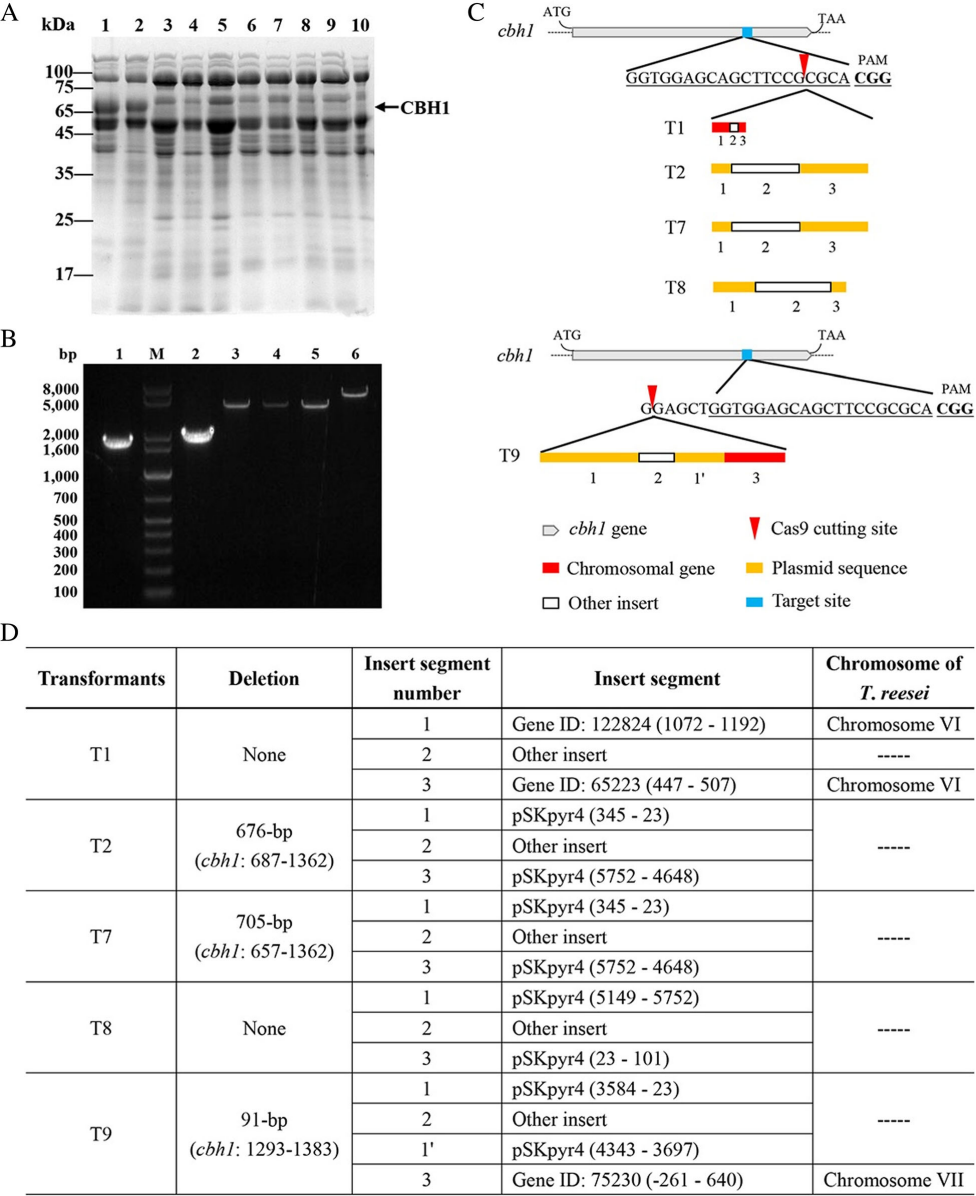
*cbh1* disruption in *T. reesei* by direct transformation of Cas9/gRNA complex and a plasmid containing the *pyr4* selection marker. A: SDS-PAGE analysis of the fermentation supernatants of the transformants. M: protein molecular mass marker, lanes 1–2: TU-6; lanes 3–11: transformants (T1, T2, T3, T4, T6, T7, T8, and T9, respectively) that did not express CBH1; B: agarose gel electrophoresis of the PCR products amplifying the *cbh1* locus from the transformants that did not express CBH1. M: DNA molecular mass marker; lane 1: TU-6; lane 2–6: the transformants T1, T2, T7, T8, and T9, respectively; C: Schematic diagram showing the inserted DNA fragments in the edited *cbh1* locus; D: Characteristics of inserted or deleted DNA fragments. The numbers for the *T. reesei* genes were counted from the start codon

Twenty-seven transformants were obtained in two transformations of Cas9/gRNA with pSKpyr4 in TU-6. Through SDS-PAGE analysis, it was suggested that eight among the 27 transformants could have the *cbh1* gene disrupted since the band corresponding to CBH1 disappeared (Fig. 3A). Interestingly, in these strains, expression of other proteins apparently increased (Fig. 3A), similar to improved secretion of a heterologous lipase in a *cbh1*-silenced *T. reesei* strain [13]. Using the primers specific for *cbh1* (Additional file 1: Table S1), a DNA fragment of 1676-bp was amplified from the parent strain TU-6 (Fig. 3B), as well as from the nineteen transformants still secreting the CBH1 protein (data not shown). Sequencing of all these DNA fragments from these transformants indicated no mutation in the *cbh1* gene. Using the same PCR condition, no DNA fragment corresponding to the 1676-bp could be amplified from the transformants (except T1) that did not secret CBH1. It has been pointed out that in *Nodulisporium* sp., transformation of a linear marker plasmid into the *cas9*-expressing host cell leads to insertion of the marker gene in the gRNA targeting locus [14]. It was hypothesized that similar integration of large DNA fragments could take place. Therefore, the PCR condition was modified, mainly by lengthening the extension time, which would allow amplification of larger DNA fragments. Using this method, we successfully amplified DNA fragments from five transformants (Fig. 3B). The sizes of the DNA fragments from four transformants (T2, T7, T8, and T9) were from 5- to 8-kb, while that for one transformant (T1) was 1.9-kb.

Further sequence analyses indicated that there were gene editing events at the expected *cbh1* locus. Deletion of 91~705-bp in *cbh1* was observed in T2, T7, and T9 (Fig. 3D). The inserted fragments varied in length and sequence by containing: i) the transformed plasmid (T2, T7, and T8); ii) a chromosome fragment (T1); or iii) mixed chromosome and plasmid sequences (T9, Fig. 3C&D). Although T2 and T7 had different deletions, they had the same inserts. It seemed that the pSKpyr4 DNA fragments starting from nucleotide 23 were favored in these gene rearrangement events (Fig. 3D). The underlying reason for this is not known. All five had another fragment (“other insert” in Fig. 3C&D) which was not from pSKpyr4 or *T. reesei* chromosome but from *E. coli* chromosome (T1) or an unidentified plasmid(s) (T2, T7, T8, and T9), which was likely contaminants from pSKpyr4 preparation.

## Discussion

The intracellularly expressed Cas9 successfully disrupts the *ura5* gene in the *T. reesei* QM6a and RUT-C30 strains by creating small deletions [7]. However, disruption of the same gene was quite different in QM9414 in this study since no such small deletions were observed in the 5-FOA-resistant transformants. In contrast, insertion (or less frequently, deletion) of small DNA fragments was found downstream of the genomic locus expected for editing. These mutations resulted in 3–4 amino acids duplication (or 3 amino acids deletion) in the *ura5*-encoded orotate phosphoribosyl transferase, which might interfere with the normal function of this enzyme, thereby enabling the transfromants to resist the toxicity of 5-FOA. Since the sequence of gRNA was the same as that used in [7], the off-target genome editing was unexpected. In the previous report [7], QM6a and RUT-C30 were used as the *cas9*-recipient strains. Being cellulase hyperproducer derivatives of QM6a, RUT-C30 and QM9414 are both created by chemical and/or physical mutations but belong to different lineages [15]. It is conceivable that the genetic backgrounds of QM9414 and the two strains (QM6a and RUT-C30) are thus quite different. It is also well-known that cellular factors involved in DNA damage response [16], NHEJ, and HDR [17] affect the CRISPR/Cas9-mediated genome editing. Therefore, it is possible that the much different genetic backgrounds may account for the unexpected genome editing.

In contrast, direct transformation of the Cas9-gRNA complex into *T. reesei* TU-6, a uridine auxotrophic mutant of QM9414, led to genome editing in the predicted *cbh1* locus. However, large DNA fragments including the co-transformed plasmid, chromosomal DNA, or their hybrid form, were found to be inserted in the targeted *cbh1* gene. Insertion of large DNA fragments was not observed in *Mucor circinelloide* [9] and *Fusarium oxysporum* [11] when direct transformation of Cas9/gRNA was employed. For these two fungi, only the Cas9/gRNA complex, but none exogenous DNA, was used in the transformation. In *Nodulisporium* sp., similar insertion of large DNA fragment was also observed. However, in that case an exogenous plasmid containing the selection marker was co-transformed with the in vitro transcribed gRNA into a recipient strain with in vivo expressed Cas9. Note, however, unlike the discovery reported herein, only the exogenous plasmid sequence was found in the inserted fragments in *Nodulisporium* sp. The promiscuity of the inserted fragments in *T. reesei* suggested complex DNA-repair events in the target locus.

With the success in *cbh1* disruption, direct transformation of Cas9/gRNA complex provides a fast means for disruption of genes of interest in *T. reesei*. This method may also be used for HDR-mediated gene replacement. In a preliminary experiment we have observed gene replacement at the *cel3c* locus, albeit at a low frequency (5 out of 143 transformants), by co-transformation of Cas9/gRNA (targeting *cel3c* [18]) with a donor DNA in *T. reesei* (data not shown). Gene replacement efficiency can be improved by optimizing the amounts, nature and ratio of Cas9 and gRNA or using a strain with dysfunctional NHEJ DNA repair pathway.

## Conclusions

The intracellularly expressed Cas9 led to biased off-target editing in *ura5*, which might be explained by the different strains used in our study and by other researchers. Direct transformation of Cas9/gRNA complex allowed disruption of *cbh1* in *T. reesei* with large DNA inserts found in the edited locus. Compared with the previous report, our method avoids the time and labor needed for introducing the *cas9* gene into *T. reesei* and the uncertainty that may occur with its expression. In our hands, both in vivo expressed Cas9 and in vitro assembled Cas9/gRNA led to insertion of small or large DNA fragments during genome editing. This might be explained by the robust NHEJ process, which is the dominant DNA repair mechanism in *T. reesei*.

## Methods

### Strains, plasmids and culture conditions

The *Escherichia coli* Trans 1-T1 strain (Transgen, Beijing, China) was used as the host for plasmid construction and propagation throughout the study. The *Saccharomyces cerevisiae* AH109 strain (Clontech, San Francisco, CA) was used for construction of the *cas9*-expressing plasmid. The pPdc1-Cas9 plasmid for expressing the codon-optimized *Streptococcus pyogenes cas9* [7] was obtained by assembling *cas9* between the strong, constitutive *pdc1* promoter and *pdc1* terminator via DNA assembler using pRS424 (New England Biolabs, Beverly, MA) as a backbone plasmid [19]. The *T. reesei* QM9414 and its uridine auxotrophic strain TU-6 were maintained in our lab. *T. reesei* was grown in the minimal medium (MM, containing (NH_4_)_2_SO_4_, 5.0 g/L; KH_2_PO_4_, 15 g/L; MgSO_4_, 0.6 g/L;CaCl_2_, 0.6 g/L; FeSO_4_·7H_2_O, 0.005 g/L; MnSO_4_·H_2_O, 0.0016 g/L; ZnSO_4_·7H_2_O, 0.0014 g/L; CoCl_2_, 0.002 g/L) supplemented with a certain kind of carbohydrate (2% glucose for mycelial growth and 2% Avicel cellulose for cellulase induction) as the sole carbon source. For sporulation, *T. reesei* was grown on potato dextrose agar (PDA) plates at 28 °C.

### Designing gRNA targeting *chh1*

The gene *cbh1* encoding the major cellulase cellobiohydrolase I (CBH1) was amplified from the genomic DNA of TU-6 by PCR using the primer pair Pcbh1F/R (Additional file 1: Table S1). It was then ligated into pEASY-T3 (Transgen, Beijing, China) to obtain pT3cbh1. Three gRNA sequences for *cbh1* were designed using the online E-CRISPR design server (http://www.e-crisp.org/E-CRISP/). The corresponding encoding DNAs were synthesized in the form of single-stranded, complementary oligonucleotides (Additional file 1: Table S1). The T7 promoter (5’-TAATACGACTCACTATAGG-3′) was designed upstream of the gRNAs. Equal amounts (1 μg) of two complementary oligonucleotides were mixed, boiled for 10 min, and cooled down at room temperature for annealing. Using the annealed double-stranded DNAs as the template, gRNAs were obtained by in vitro transcription with a MEGAshortscript T7 Transcription Kit (Invitrogen, Carlsbad, CA) and the RNAs were purified by using the RNA Purification Kit (Tiangen, Beijing, China). To determine the ability to guide digestion, 1 μg of gRNA, 2 μg of recombinant Cas9 (GenScript, Nanjing, China), and 200 ng of the *Bcu*I-linearized pT3cbh1 plasmid were mixed and incubated at 37 °C for 2 h. At the end of reaction, the samples were taken out for agarose gel electrophoresis analysis.

### Transformation of *T. reesei*

For plasmid transformation, pPdc1-Cas9 and pRLMex30 (carrying the hygromycin B phosphotransferase gene) were co-introduced into the *T. reesei* QM9414 strain by polyethylene glycol (PEG)-mediated chemical transformation [20]. The transformants were selected on MM-glucose plates containing 150 μg/ml of hygromycin. For transformation of gRNA targeting *ura5*, 6.5 μg of in vitro transcribed gRNA were similarly introduced into *T. reesei* through the same PEG-mediated protoplast transformation method. MM-glucose supplemented with 5-fluoroorotic acid (5-FOA, 3 mg/ml) and 10 mM uridine was used to select the transformants. Expression of wild-type, functional *ura5* (encoding orotate phosploribosyl transferase) will convert 5-FOA into toxic 5′-fluorouridine monophosphate. For Cas9/gRNA transformation, 12 μg of the Cas9 protein and 6.5 μg of gRNA were mixed and incubated at 37 °C for 30 min for in vitro assembly into the Cas9/gRNA RNP complex. The complex was then co-transformed with 6 μg of the pSKpyr4 plasmid, which contains the *pyr4* marker gene expression cassette [13], into the *T. reesei* TU-6 protoplasts.

### Analyzing *cbh1* disruption in the transformants

The spores (1 × 10^8^) of TU-6 and its transformants were individually inoculated into 100 ml of liquid MM-glucose (2%) and cultured at 28 °C with shaking for 48 h. Mycelia were collected by filtering through a 200-mesh sifter, washed with MM, and transferred into the MM-Avicel cellulose (2%) medium to induce cellulase expression. The fermentation broth on the eighth day after cellulose induction was analyzed by SDS-PAGE. The genomic DNAs of TU-6 and its transformants were extracted from the harvested mycelia and were used as templates for PCR amplification of the *cbh1* locus. The PCR conditions were 94 °C for 1 min for initial denaturation, then 24 cycles including 94 °C for 25 s, 61 °C for 30 s (temperature reduced by 0.2 °C per cycle), and 70 °C for 5 min.

### Gene replacement of *cel3c*

The Cas9 (12 μg) was in vitro assembled with 6.5 μg of gRNA targeting *cel3c* (GenBank accession number: AY281375.1) by incubation at 37 °C for 30 min. The donor DNA was consisted of two homology arms (1.0 kb each) flanking the *cel3c* locus. The homology arms were intercepted by an expression cassette for *Melanocarpus albomyces* laccase (GenBank accession number: CAE00180, under the control of *cbh1* promoter and terminator) and the *pyr4* marker gene. The Cas9/gRNA complex and 3 μg of the donor DNA were co-transformed into *T. reesei* protoplasts and the transformants were transferred into MM-lactose plate containing 0.4 mM ABTS and 0.1 mM CuSO_4_. Laccase oxidizes ABTS and blue halos form around the laccase-expressing colonies. Laccase-positive transformants were grown on PDA plates for sporulation. Replacement of *cel3c* in these transformants was investigated using diagnostic PCR amplifying the fragments traversing across the chromosome and the *cel3c* editing locus.

## Additional file

Additional file 1: **Table S1.** Primers used in this study. **Figure S1.** Sequencing the *ura5* gene of QM9414, C5 expressing Cas9 intracellularly, and the C5-derived transformants. (DOCX 1220 kb)

## Abbreviations

5-FOA: 5-fluoroorotic acid
CBH1: cellobiohydrolase I
CRISPR/Cas9: Clustered Regularly Interspaced Short Palindromic Repeats/CRISPR associated protein 9
HDR: homology-based DNA recombination
MM: minimal medium
NHEJ: non-homologous end-joining
PDA: potato dextrose agar
PEG: polyethylene glycol
RNP: ribonucleoprotein
