# Supplementary material for: Fast gene disruption in Trichoderma reesei using in vitro assembled Cas9/gRNA complex
Source: BMC Biotechnol. 2019 Jan 9;19:2. doi: 10.1186/s12896-018-0498-y (PMC6325762; doi:10.1186/s12896-018-0498-y)
Supplement: Supplementary file 1 — Table S1. Primers used in this study. Figure S1. Sequencing the ura5 gene of QM9414, C5 expressing Cas9 intracellularly, and the C5-derived transformants. (DOCX 1220 kb) [file 12896_2018_498_MOESM1_ESM.docx]

**Additional file 1**

**Table S1. Primers used in this study**

| **Primer** | **Sequence (5′–3′)** | **Usage** |
| --- | --- | --- |
| Pcbh1F | ATGTATCGGAAGTTGGCCGTCATCT | Amplification of *cbh1* for construction of pT3cbh1 |
| Pcbh1R | TTACAGGCACTGAGAGTAGTAAGGG | Amplification of *cbh1* for construction of pT3cbh1 |
| M13F | GTAAAACGACGGCCAGT | Screening *E. coli* transformants for positive pT3cbh1 |
| M13R | GTCATAGCTGTTTCCTG | Screening *E. coli* transformants for positive pT3cbh1 |
| gRNA1(cbh1)F | aagcTAATACGACTCACTATAGGGCTGGCGCCAAGTACGGCACgttttagagctagaaatagcaagttaaaataaggctagtccgttatcaacttgaaaaagtggcaccgagtcggtgctttt | *In vitro* transcription of gRNA1(cbh1) |
| gRNA1(cbh1)R | AAAAGCACCGACTCGGTGCCACTTTTTCAAGTTGATAACGGACTAGCCTTATTTTAACTTGCTATTTCTAGCTCTAAAACGTGCCGTACTTGGCGCCAGCCCTATAGTGAGTCGTATTAGCTT | *In vitro* transcription of gRNA1(cbh1) |
| gRNA2(cbh1)F | aagcTAATACGACTCACTATAGGGCTGAGGAGGCAGAATTCGGgttttagagctagaaatagcaagttaaaataaggctagtccgttatcaacttgaaaaagtggcaccgagtcggtgctttt | *In vitro* transcription of gRNA2(cbh1) |
| gRNA2(cbh1)R | AAAAGCACCGACTCGGTGCCACTTTTTCAAGTTGATAACGGACTAGCCTTATTTTAACTTGCTATTTCTAGCTCTAAAACCCGAATTCTGCCTCCTCAGCCCTATAGTGAGTCGTATTAGCTT | *In vitro* transcription of gRNA2(cbh1) |
| gRNA3(cbh1)F | aagcTAATACGACTCACTATAGGGGTGGAGCAGCTTCCGCGCAgttttagagctagaaatagcaagttaaaataaggctagtccgttatcaacttgaaaaagtggcaccgagtcggtgctttt | *In vitro* transcription of gRNA3(cbh1) |
| gRNA3(cbh1)R | AAAAGCACCGACTCGGTGCCACTTTTTCAAGTTGATAACGGACTAGCCTTATTTTAACTTGCTATTTCTAGCTCTAAAACTGCGCGGAAGCTGCTCCACCCCTATAGTGAGTCGTATTAGCTT | *In vitro* transcription of gRNA3(cbh1) |
| gRNA(ura5)F | aagcTAATACGACTCACTATAGGGGCGAGGGCGGCAACATCGTGTTTTAGAGCTAGAAATAGCAAGTTAAAATAAGGCTAGTCCGTTATCAACTTGAAAAAGTGGCACCGAGTCGGTGCTTTT | *In vitro* transcription of gRNA(ura5) |
| gRNA(ura5)R | AAAAGCACCGACTCGGTGCCACTTTTTCAAGTTGATAACGGACTAGCCTTATTTTAACTTGCTATTTCTAGCTCTAAAACACGATGTTGCCGCCCTCGCCCCTATAGTGAGTCGTATTAGCTT | *In vitro* transcription of gRNA(ura5) |
| gRNA(cel3c)F | AAGCTAATACGACTCACTATAGGGATGGAAGGACTGGATGACAGTTTTAGAGCTAGAAATAGCAAGTTAAAATAAGGCTAGTCCGTTATCAACTTGAAAAAGTGGCACCGAGTCGGTGCTTTT | *In vitro* transcription of gRNA(*cel3c*) |
| gRNA(cel3c)R | AAAAGCACCGACTCGGTGCCACTTTTTCAAGTTGATAACGGACTAGCCTTATTTTAACTTGCTATTTCTAGCTCTAAAACTGTCATCCAGTCCTTCCATCCCTATAGTGAGTCGTATTAGCTT | *In vitro* transcription of gRNA(*cel3c*) |
| yz-Pura5F | ATGGCTACCACCTCCCAGCTGCCTG | Amplification of *ura5* gene |
| yz-Pura5R | TCAGTCAGTCGCCTTGTACTTGGCA | Amplification of *ura5* gene |
| RTQactF | TGAGAGCGGTGGTATCCACG | RT-qPCR for *actin* |
| RTQactR | GGTACCACCAGACATGACAATGTTG | RT-qPCR for *actin* |
| RTQcas9F | cgaggaagacaagaagcacg | RT-qPCR for *cas9* |
| RTQcas9R | gccgcggaacttaatcatgt | RT-qPCR for *cas9* |
| TrPpdc1pF | ATAAGCTTGATATCGAATTCGTGTCGAGCCGGGAGGAGTT | Cloning *pdc1* promoter for pPdc1-Cas9 construction |
| TrPpdc1pR | TCATGCCGGGctgcagGATTGTGCTGTAGCTGCGC | Cloning *pdc1* promoter for pPdc1-Cas9 construction |
| TrPpdc1tF | AATCctgcagCCCGGCATGAAGTCTGACCG | Cloning *pdc1* terminator for pPdc1-Cas9 construction |
| TrPpdc1tR | GAACTAGTGGATCCCCCGGGCGTCGGCCGGGTGGTGAGCT | Cloning *pdc1* terminator for pPdc1-Cas9 construction |
| yz-Ppdc1F | gtcaccagtataaataaccgcatca | Screening *T. reesei* transformants for positive pPdc1-Cas9 |
| yz-PCas9R | aggggaccttgtactcgtccgtg | Screening *T. reesei* transformants for positive pPdc1-Cas9 |
| TrPcbh1F | aaaacgatcaaagcagcgcagctacagcacaatcatggacaagaagtacagcattggcc | Amplification of *cas9* for pPdc1-Cas9 construction |
| TrPcbh1R | accctcatactacccggtcagacttcatgccgggttagaccttgcgcttcttcttgggg | Amplification of *cas9* for pPdc1-Cas9 construction |
| yz-Pcbh1F | ATGTATCGGAAGTTGGCCGTCATCT | Amplification of *cbh1* locus in Cas9/gRNA tranformants |
| yz-Pcbh1R | TTACAGGCACTGAGAGTAGTAAGGG | Amplification of *cbh1* locus in Cas9/gRNA tranformants |
| yz-cel3cF | GGTATCAAGTTGTGATGATGAAGCTGACAGCGG | Verifying gene replacement at the *cel3c* locus |
| yz-cel3cR | CTGAGGCCGTCAAAGGGAGTGACTGC | Verifying gene replacement at the *cel3c* locus |


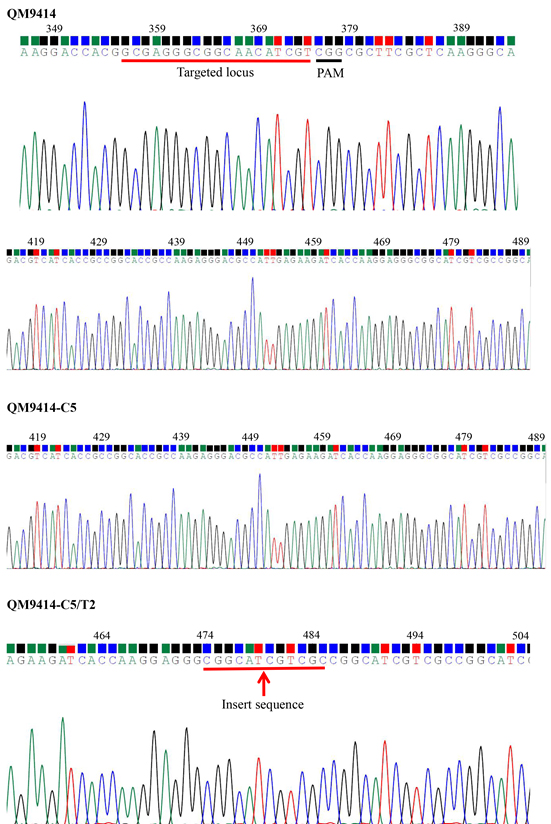


**Figure S1**. Sequencing the *ura5* gene of QM9414, C5 expressing Cas9 intracellularly, and the C5-derived transformants.

**
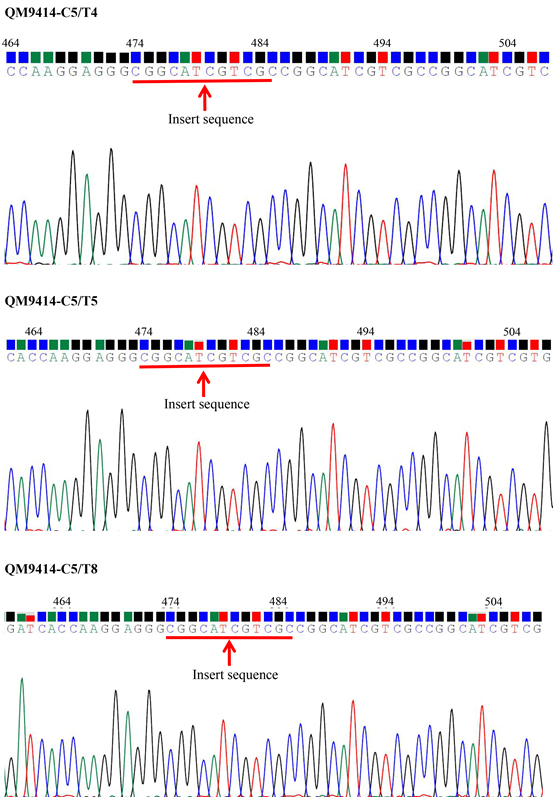
**

**Figure S1 (continued)**. Sequencing the *ura5* gene of QM9414, C5 expressing Cas9 intracellularly, and the C5-derived transformants.

**
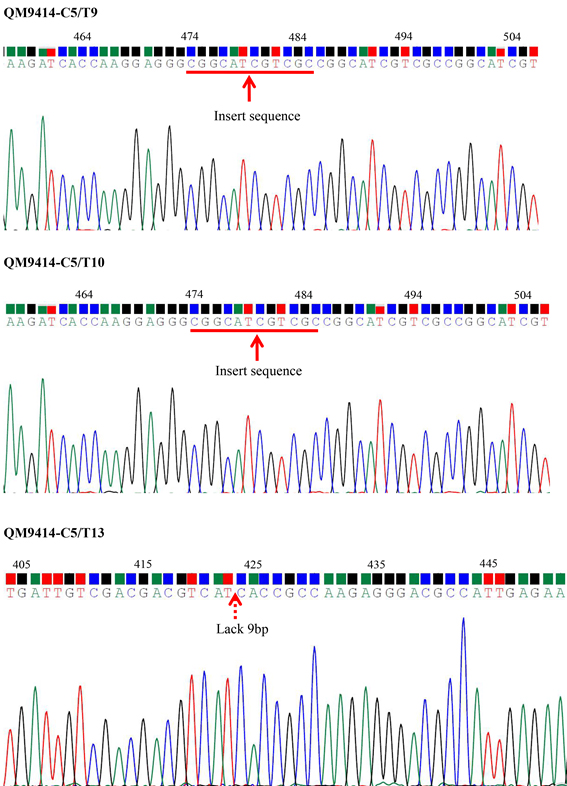
**

**Figure S1 (continued)**. Sequencing the *ura5* gene of QM9414, C5 expressing Cas9 intracellularly, and the C5-derived transformants.

**
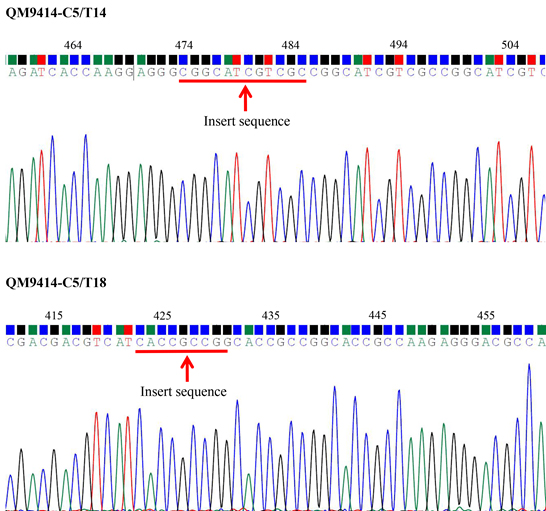
**

**Figure S1 (continued)**. Sequencing the *ura5* gene of QM9414, C5 expressing Cas9 intracellularly, and the C5-derived transformants.
